# Healthcare Resource Utilization among Patients between 60–75 Years with Secondary Acute Myeloid Leukemia Receiving Intensive Chemotherapy Induction: A Spanish Retrospective Observational Study

**DOI:** 10.3390/cancers14081921

**Published:** 2022-04-11

**Authors:** Antonio Solana-Altabella, Juan Eduardo Megías-Vericat, Octavio Ballesta-López, Blanca Boluda, Isabel Cano, Evelyn Acuña-Cruz, Rebeca Rodríguez-Veiga, Laura Torres-Miñana, Claudia Sargas, Miguel Á. Sanz, Carmela Borrell-García, Eduardo López-Briz, José Luis Poveda-Andrés, Javier De la Rubia, Pau Montesinos, David Martínez-Cuadrón

**Affiliations:** 1 Pharmacy Department, Hospital Universitari i Politècnic La Fe, 46026 Valencia, Spain; solana_ant@gva.es (A.S.-A.); megias_jua@gva.es (J.E.M.-V.); ballesta_oct@gva.es (O.B.-L.); borrell_car@gva.es (C.B.-G.); lopez_edubri@gva.es (E.L.-B.); poveda_josand@gva.es (J.L.P.-A.); 2 Hematology and Hemotherapy, Instituto de Investigación Sanitaria La Fe, 46026 Valencia, Spain; boluda_bla@gva.es (B.B.); cano_isafer@gva.es (I.C.); evelyn_acuna@iislafe.es (E.A.-C.); rodriguez_reb@gva.es (R.R.-V.); laura_torres@iislafe.es (L.T.-M.); claudia_sargas@iislafe.es (C.S.); miguel.sanz@uv.es (M.Á.S.); delarubia_jav@gva.es (J.D.l.R.); montesinos_pau@gva.es (P.M.); 3 Hematology Department, Hospital Universitari i Politècnic La Fe, 46026 Valencia, Spain; 4 School of Medicine and Dentistry, Catholic University of Valencia, 46010 Valencia, Spain

**Keywords:** health care costs, secondary acute myeloid leukemia, hospitalization, reimbursement

## Abstract

**Simple Summary:**

Studies addressing the economic costs and burden of secondary acute myeloid leukemia (sAML) are scarce in the literature. We analyzed this topic in a real-life population of sAML patients between 60–75 years receiving intensive chemotherapy induction. In elderly patients with sAML and intensive regimens, it entails an increase in costs and a longer hospital stay. In these specific patients, almost a third of the time is spent hospitalized after the diagnosis of sAML. There are no studies with this type of population and diagnosis, which gives added value to the results obtained. Pharmacoeconomic studies in patients with AML are being carried out due to the need to evaluate the cost-effectiveness of new oral drugs, therapeutic schemes with higher costs than previous treatments.

**Abstract:**

Background: Information regarding the impact on healthcare systems of secondary acute myeloid leukemia (sAML) is scarce. Methods: A retrospective review of medical charts identified patients aged 60–75 years with sAML between 2010 and 2019. Patient information was collected from diagnosis to death or last follow-up. Outpatient resource use, reimbursement, frequency and duration of hospitalization, and transfusion burden were assessed. Forty-six patients with a median age of 64 years were included. Anthracycline plus cytarabine regimens were the most common induction treatment (39 patients, 85%). The ratio of the total days hospitalized between the total follow-up was 29%, with a sum of 204 hospitalizations (average four/patient; average duration 21 days). The total average reimbursement was EUR 90,008 per patient, with the majority (EUR 77,827) related to hospital admissions (EUR 17,403/hospitalization). Most hospitalizations (163, mean 22 days) occurred in the period before the first allogeneic hematopoietic stem cell transplant (alloHSCT), costing EUR 59,698 per patient and EUR 15,857 per hospitalization. The period after alloHSCT (in only 10 patients) had 41 hospitalizations (mean 21 days), and a mean reimbursement cost of EUR 99,542 per patient and EUR 24,278 per hospitalization. In conclusion, there is a high consumption of economic and healthcare resources in elderly patients with sAML receiving active treatments in Spain.

## 1. Introduction

Secondary acute myeloid leukemia (sAML) includes a heterogeneous group of patients with an antecedent hematologic disorder or acute myeloid leukemia related to treatment (tAML), mainly chemotherapy (CHT) and/or radiotherapy [1,2]. The reported relative incidence of sAML ranges from 18% to 28% of all AML cases [1,3,4,5,6]. Furthermore, sAML has usually been associated with a worse prognosis [3,4,5], including a lower rate of complete remission (CR), overall survival (OS), and relapse-free survival (RFS) than de novo AML [7,8,9,10,11,12,13,14]. sAML patient features include poor prognosis factors such as older age, comorbidities/organ dysfunctions, worse performance status (ECOG ≥2), and adverse cytogenetic and molecular profiles [4,15,16,17,18]. Indeed, the diagnosis of sAML has been considered an independent risk factor for early death and shorter OS in some predictive models [15,16,18].

The emergence of new treatments for sAML, such as CPX-351, could lead to improved CR and OS as compared to the standard 7 + 3 scheme of anthracyclines and cytarabine (Ara-C) [19,20]. CPX-351 is a fixed 5:1 molar ratio of Ara-C and daunorubicin which has been approved for adults with tAML or AML with myelodysplasia-related changes (MRC) by the Food and Drug Administration (FDA) and European Medicines Agency (EMA), and has been included in ELN and NCCN guidelines [21,22]. It should be noted that novel therapies for hematological malignancies, such as CPX-351, are leading to an incremental pharmaceutical cost in the treatment of patients. In this context, the outcome of the following study is to establish the baseline costs and healthcare resource utilization (HCRU) of sAML subjects managed under standard approaches. These data could be useful for the assessment of the pharmacoeconomic impact of CPX-351 in this setting.

## 2. Materials and Methods

### 2.1. Patients

Patients consecutively diagnosed with tAML or AML-MRC between 2010 and 2019 in a single tertiary care institution (Hospital Universitari I Politècnic La Fe, Valencia, Spain) were identified. Patients eligible for inclusion in the study were aged 60 to 75 years old and were hospitalized for the management of at least one episode of tAML or AML-MRC, according to WHO 2016 classification [2]. All patients were treated using intensive front-line schedules.

### 2.2. Study Design

Single-center, retrospective study, performed from 2010 to 2019. The protocol was approved by the local Clinical Research Ethics Committee, in accordance with the principles of the Declaration of Helsinki.

From the date of diagnosis of tAML or AML-MRC to death or last follow-up, individual patient data were collected. The index date from diagnosis was considered and was defined as the first date on which the tAML or AML-MRC diagnosis was recorded in the clinical history. The cut-off date for the analysis was December 2019.

The CHT period was defined as the time from the index date to loss to follow-up, initiation of allogeneic hematopoietic stem cell transplantation (alloHSCT), or death. If the induction treatment was alloHSCT, the patient was not included in the analysis of the CHT period.

The alloHSCT period was defined as the time from hospitalization for alloHSCT to loss to follow-up or death.

### 2.3. Outcomes

Outpatient resource use, reimbursement, frequency and duration of hospitalization, and transfusion burden were assessed as primary outcomes. Secondary outcomes included description of tAML or AML-MRC patients: demographic, clinical, and treatment at induction. sAML-related HCRU was analyzed among a selected cohort of patients, including frequency, type, and duration of hospitalizations; reasons for hospitalizations; and reimbursement for hospitalizations, outpatient resources such as hospital day visits or clinic visits, and transfusions. The different variables were analyzed for both periods (CHT and alloHSCT). Reasons for admission were recorded for each hospitalization (there could be more than one reason for admission).

### 2.4. Calculation of Hospital Reimbursement

**Figure 1:**
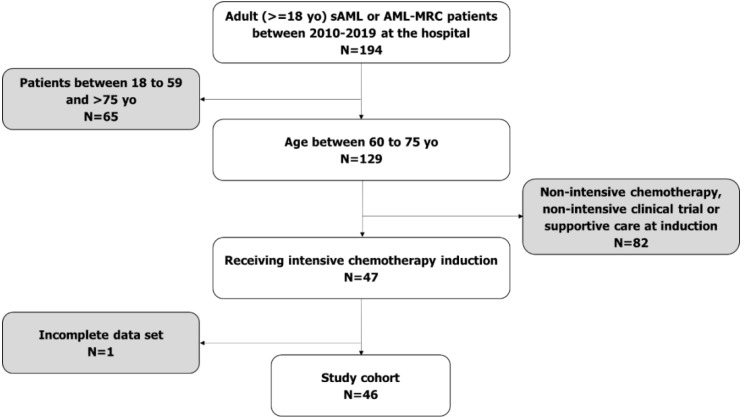
Decision algorithm for assignment of DRG code for hospitalization episodes. yo: years old, sAML: secondary acute myeloid leukemia, AML-MRC: acute myeloid leukemia with myelodysplasia-related changes.

The list of national groups related to diagnosis (DRG) allows the standardized calculation of the amount of money reimbursed to the hospital and the length of hospitalization for each DRG code. DRG cost at the Spanish Health System encompassed direct, semi-direct, indirect, and structural costs (cost of drugs and personnel are included). These costs were classified into three groups: personnel costs, costs of goods sold and services, and amortization expense, and assigned relative to each pathology of similar patients [23,24]. The reimbursement followed the algorithm for hospital admissions. Reimbursement was established for ambulatory care at EUR 108 per visit and for each day hospital stay at EUR 269. These values were calculated with the average costs of day hospital units in Spain [25]. Each DRG code (2016) and its associated reimbursement was assigned for each hospitalization (Figure 1) [26]: DRG 576 (EUR 27,274) if intensive induction schemes were used; DRG 577 (EUR 12,444) if intensification or consolidation schemes were used; DRG 577 (EUR 12,444) if there was a major complication (for example, parenteral nutrition, pneumonia, respiratory failure, or sepsis) even if there was no CHT treatment; DRG 876 (EUR 4475) if admission was not related to intensive CHT and no major complication had occurred. DRG code 803 was assigned to the admission in which alloHSCT is performed, with a high reimbursement of EUR 60,599. In that admission and later, if the admission exceeds 40 days, EUR 692 is added for each extra day of hospitalization [25].

Major complications were associated and the DRG code 577 was used in some hospitalizations in which the length of stay was much longer than the average provided in the DRG list.

### 2.5. Statistical Analyses

Based on the experience with this rare disease in the hospital, an adequate sample size of 50 patients was established. All patients who met the inclusion criteria were included. Quantitative variables were expressed as mean and standard deviation (SD), or confidence interval (CI), or median and Interquartile Range (IQR). Categorical variables were shown with frequency and percentage. Statistical analysis was performed using Stata 14.2 software.

## 3. Results

### 3.1. Patients

**Figure 2:**
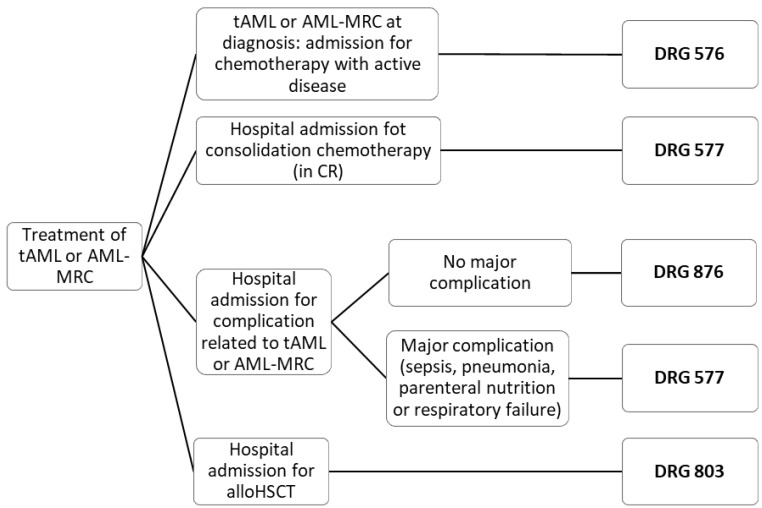
Flowchart diagram (patient enrolment). DRG: diagnosis-related group, tAML: acute myeloid leukemia related to treatment, AML-MRC: acute myeloid leukemia with myelodysplasia-related changes, CR: complete remission, alloHSCT: allogeneic hematopoietic stem cell transplantation.

Forty-six patients with sAML (tAML or AML-MRC) were eligible for inclusion in the study, following exclusion of one ineligible case (Figure 2). The median age was 64 years and 74% of patients were men (Table 1). The median follow-up was 328 days (overall 15,066 days of follow-up/exposure for the entire cohort). Overall, 204 admissions occurred during the study period, and 4382 days were spent in hospital. The inpatient life vs. outpatient life ratio was 0.29.

Response to induction CHT was 23 (50%) CR or incomplete CR (CRi), 15 (33%) partial remission or resistance, and 8 (17%) induction death (at any point during induction).

For this part of the analysis, we considered the HCRU across all the study periods, from the index date until the last follow-up. We observed that sAML therapy was associated with a mean per patient hospitalization of 95 days, with a mean overall reimbursement per patient of EUR 90,008. In Table 2, we show the days of exposure across the study period. Thirty-six patients (78%) had died by the end of the study period.

### 3.2. Hospitalizations and Reimbursement during the Chemotherapy Period

Forty-five patients received intensive schemes and one patient received an alloHSCT directly in induction. Therefore, 44 patients were included in the CHT period. The mean time of patients in the CHT period was 200 (SD 287) days, in which there were a total of 163 hospitalizations, mean duration of 22 days per hospitalization, and each patient had 4 hospitalizations on average (Table 3). In the CHT period, the ratio of hospital stay/total time was 58% (95% confidence interval (CI): 50–67%). The mean total cost was EUR 67,269 (SD EUR 47,824) per patient, most of which was related to hospitalization (EUR 59,698; 89%), with a reimbursement of EUR 15,857 per hospitalization. The mean number of external visits and day hospital visits accounted for 20 visits each (Table 3).

### 3.3. Hospitalizations and Reimbursement for Patients who Underwent alloHSCT

Ten patients underwent alloHSCT (Table 4). During the alloHSCT time period, there were 41 inpatient hospitalizations with a mean length of hospital stay similar (21 days) to the CHT treatment period, but inpatient hospitalization reimbursement was higher (EUR 24,278) during alloHSCT period. Patients spent a mean of 42% (95% CI: 28–55%) of time hospitalized during alloHSCT period, and alloHSCT hospitalizations were associated with a mean reimbursement of EUR 99,542 per patient and an overall reimbursement of EUR 123,760 (Table 4). Five patients had died by the end of the alloHSCT period.

### 3.4. Reasons for Hospitalizations

**Figure 3:**
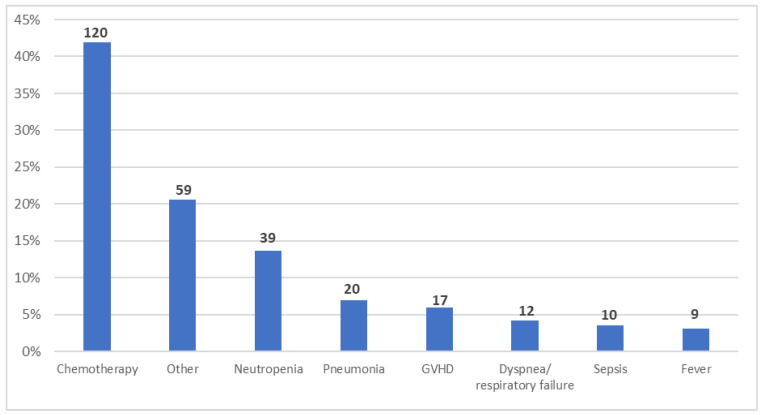
Reasons for inpatient hospitalization during the study period (*n* = 286). Reasons with a frequency of 3% or more are shown; all others are grouped together in the “Other” category. Patients could be hospitalized for more than one reason. GVHD: graft-versus-host disease.

The most common primary reasons for hospitalizations were CHT administration (42%), febrile neutropenia (14%), pneumonia (7%), and graft-versus-host disease (GVHD) (6%) (Figure 3). If the frequency of the reason for admission was less than 3%, it was not included separately in the analysis and was assigned as “Other” (Figure 3).

## 4. Discussion

Our study shows that the current standard treatment of tAML and AML-MRC is associated with prolonged hospitalizations (95 days per patient) and high costs (EUR 90,008 overall cost) at a Spanish hospital. Most of tAML and AML-MRC hospitalizations were associated with CHT administration and/or major complications, which means higher DRG associated costs. Once diagnosed with sAML, adult patients spent almost a third of their time in the hospital. More granular HCRU and economic data are needed in sAML patients treated with standard therapies in order to conduct an HCRU comparison between standard therapies and novel agents.

In this study, we analyzed tAML and AML-MRC together, characterized as AML patients because their clinical management, treatment, and prognosis are similar. Furthermore, novel therapies, such as CPX-351, are approved for tAML and AML-MRC. We selected patients 60–75 years old because they fit with the inclusion criteria of the pivotal phase three trial leading to CPX-351 approval. Regarding causes of hospital admission, in our cohort the most common were CHT and neutropenia. The goal in the sAML setting is to first reach a CR/CRi in order to perform an alloHSCT. The rate of CR/CRi in our cohort was 50% (23 out of 46); however, we found that only 10 patients (22%) received an alloHSCT, mainly due to their clinical situation and previous state. Costs associated with alloHSCT were higher compared with patients without HSCT, in alignment with the findings of two recently published studies in de novo AML [25,27].

HCRU studies in AML are relatively scarce, and are rarely analyzed in subpopulations such as sAML patients [28]. Given the torpid evolution of patients with sAML compared to patients with de novo AML, it is relevant to assess HCRU, as well as the time and cost of hospitalization, to achieve a global vision that allows us to define the therapeutic place of new treatments.

Thus far, the majority of pharmacoeconomic studies in AML have been conducted in the United States of America (US) [25,26,28,29,30,31,32], which can be considered a less cost-effective health system (14.32% of the gross domestic product (GDP), EUR 7577 per capita) compared to the Spanish healthcare system (6.24% of the GDP, EUR 1617 per capita) [33]. Two USA studies reported higher costs than our study, estimating a mean overall cost of USD 181,538 per patient in a series of 1597 patients with de novo AML or relapsed/refractory (R/R) AML [31], and USD 439,104 in 707 patients with only R/R AML [25]. Another study performed in R/R AML patients revealed costs ranging from USD 19,330 to USD 24,7840 per person/month, and a mean total cost per patient of USD 307,733 in 6415 patients [26]. Comparing these studies with our results suggests that R/R AML treatment costs, in particular in the US, are higher. In fact, a study performed by our group showed a mean cost per patient of EUR 108,293 in adult patients with R/R FLT3 mutated AML [27].

As far as we know, only two studies have specifically analyzed the economic costs and burden in sAML [28,34]. A study analyzed HCRU in a phase three trial of CPX-351 in patients with de novo high-risk/secondary AML [28]. The CPX-351 arm reported similar hospital stays and supportive care therapy (transfusion, anti-infective agents, and growth factor requirements) compared to the 7 + 3 scheme, despite a longer median length of treatment (62 vs. 41 days) related to the greater proportion of post-remission patients and higher hematologic toxicity. Unfortunately, this study did not account for the costs between both therapies. A retrospective chart review performed in the Netherlands including de novo AML and sAML patients treated intensively obtained a comparable mean overall cost to that in our series (EUR 117.495 vs. EUR 90,008) [34]. However, the comparison of these data should be taken with caution since they are not comparable methods. Arenaza et al., in a pharmacoeconomic multicenter Spanish study in de novo FLT3 positive AML patients from the RATIFY study, showed an overall cost of EUR 121,374 in non-alloHSCT patients and EUR 159,900 (no midostaurin arm) in alloHSCT patients. These higher reimbursements could be explained by the fact that in these cohorts patients were younger and probably more fit and thus could receive more treatment schemes (with hospital admissions), including R/R episodes or alloHSCT [35].

Some limitations should be addressed. First is the retrospective design of the study, since variables collected retrospectively may be less precise than variables recorded directly in the medical record. Secondly, although we used standardized cost values for Spain, these data were recorded from a single tertiary center, which could limit the external validity of the study. Finally, the use of derived variables based on DRGs could not reflect the real costs and healthcare in clinical practice, but it is an extended and validated method for obtaining homogenized and reproducible pharmacoeconomic studies. Although our study has limitations, it has advantages in that these data are unique, as they illustrate the economic impact in a real-life population of unselected sAML patients. We should highlight that in our series only 36% of elderly patients were treated with intensive CHT and only 10 patients (8%) received alloHSCT. In addition, almost 30% of their life during disease was spent in the hospital. These numbers raise questions on conventional treatments and the use of novel drugs, which could significantly vary the cost/effectiveness balance for this patient population.

## 5. Conclusions

In conclusion, there is a high consumption of economic and healthcare resources in elderly patients with tAML and AML-MRC patients receiving active treatments in Spain, but less than in other countries A balance must be found between the cost associated with new therapies, such as oral drugs, and the decrease in HCRU associated with these new treatments in patients with sAML. It would be necessary to perform comparative pharmacoeconomic studies for different treatments in the different types of AML in order to assess whether these are cost-effective and to elucidate the subset of patients which could benefit from these therapies.

## Figures and Tables

**Table 1:** Patient characteristics.

Characteristic	*n* = 46
Age at index date, years	
Median (IQR)	64 (6)
Mean (SD)	65 (4)
Men, *n* (%)	34 (74)
Status at the end of follow-up, *n* (%)	
Deceased	36 (78)
Alive	10 (22)
sAML groups, *n* (%)	
AML-MRC	31 (67)
tAML	15 (33)
Treatment received at induction, *n* (%)	
IDA + Ara-C	29 (63)
Daunorubicin + Ara-C	9 (20)
Ara-C	6 (13)
FLAG-IDA	1 (2)
alloHSCT	1 (2)
Patients in period, *n* (%)	
CHT	45 (98)
alloHSCT	10 (22)

IQR: Interquartile Range, SD: standard deviation, %: percentage of patients, sAML: secondary acute myeloid leukemia, tAML: therapy-related myeloid acute myeloid leukemia, AML-MRC: acute myeloid leukemia with myelodysplasia-related changes, IDA + Ara-C: idarubicin/cytarabine, Ara-C cytarabine, FLAG-IDA: Fludarabine/cytarabine/G-CSF/idarubicin, CHT: chemotherapy, alloHSCT: allogeneic hematopoietic stem cell transplantation.

**Table 2:** Mean and median healthcare resource utilization.

Healthcare Resource Unit	Inpatient Hospitalizations	External Consultation Visits	Day Hospital Visits	Overall
Number of hospitalization or visits	204	1229	1106	NA
Mean (SD), median [IQR] length of stay per episode, days	21 (14)	1 (0)	1 (0)	NA
	22 [21]			
Mean (SD), median [IQR] reimbursement per hospitalization or visit, EUR	17,403 (13,050)	108 (0)	270 (0) ^1^	NA
	12,444 [14,830]			
Mean (SD), median [IQR] number of stays per patient	4 (3)	27 (29)	24 (31)	NA
	4 [4]	19 [38]	17 [32]	
Mean (SD), median [IQR] days of hospitalization per patient	95 (70)	27 (29)	24 (31)	NA
	82 [105]	19 [38]	17 [32]	
Mean (SD), median [IQR] reimbursement per patient, EUR	77,827 (51,736)	2885 (3145)	9296 (11,841)	90,008 (63,413)
	68,528 [86,003]	2052 [4104]	4581 [12,562]	73,487 [102,723]
Mean (SD), median [IQR] number RBC packages transfusion per patient	27 (21)	NA	7 (12)	34 (29)
	19 [28]		2 [10]	23 [33]
Mean (SD), median [IQR] number platelet transfusion per patient	33 (29)	NA	6 (12)	38 (35)
	24 [36]		1 [7]	26 [39]

^1^ EUR 691 after allogeneic hematopoietic stem cell transplantation (alloHSCT). IQR: Interquartile Range, NA: not applicable, SD: standard deviation, RBC: Red Blood Cells.

**Table 3:** Hospitalizations and reimbursement during the chemotherapy period (previous to the first alloHSCT and in patients with no alloHSCT).

Healthcare Resource Unit	Inpatient Hospitalizations	External Consultation Visits	Day Hospital Visits
Per hospitalization			
Number of hospitalizations	163	952	799
Mean (SD), median [IQR] length of stay, days	22 (14)	1 (0)	1 (0)
	22 [22]		
Mean (SD), median [IQR] reimbursement, EUR	15,857 (9169)	108 (0)	270 (0)
	12,445 [14,830]		
Per patient			
Number of patients	45	45	45
Mean (SD), median [IQR] number of stays	4 (3)	20 (27)	20 (33)
	3 [5]	10 [26]	8 [18]
Mean (SD), median [IQR] length of stay, days	81 (62)	NA	NA
	68 [106]		
Mean (SD), median [IQR] reimbursement, EUR	59,698 (38,123)	2211 (2886)	5360 (8762)
	52,164 [56,679]	1080 [2754]	2291 [4716]

IQR: Interquartile Range, NA: not applicable, SD: standard deviation.

**Table 4:** Hospitalizations and reimbursement after alloHSCT.

Healthcare Resource Unit	Inpatient Hospitalizations	External Consultation Visits	Day Hospital Visits
Per hospitalization			
Number of hospitalizations	41	277	307
Mean (SD), median [IQR] length of stay, days	21 (15)	1 (0)	1 (0)
	19 [19]		
Mean (SD), median [IQR] reimbursement, EUR	24,278 (21,944)	108 (0)	691 (0)
	12,445 [25,612]		
Per patient			
Number of patients	10	10	10
Mean (SD), median [IQR] number of stays	4 (3)	28 (21)	31 (16)
	3 [4]	24 [29]	30 [23]
Mean (SD), median [IQR] length of stay, days	87 (71)	NA	NA
	59 [43]		
Mean (SD), median [IQR] reimbursement, EUR	99,542 (38,888)	2992 (2263)	21,226 (11,282)
	80,949 [64,608]	2646 [3132]	21,088 [15,902]

IQR: Interquartile Range, NA: not applicable, SD: standard deviation.

## Data Availability

The datasets generated and analyzed during the current study are property of the IISLAFE. For data requests, please contact martinez_davcua@gva.es.
